# The Role of FGFR3 in the Progression of Bladder Cancer

**DOI:** 10.3390/cancers17213588

**Published:** 2025-11-06

**Authors:** Sahoko Ninomiya, Yukari Ishiguro, Hisashi Hasumi, Ryosuke Jikuya, Akihito Hashizume, Masanobu Yamazaki, Jun-ichi Teranishi, Kazuhide Makiyama, Hiroji Uemura, Hiroshi Miyamoto, Takashi Kawahara

**Affiliations:** 1 Department of Urology and Renal Transplantation, Yokohama City University Medical Center, Yokohama 232-0024, Japan; t216050c@yokohama-cu.ac.jp (S.N.); y1496@yokohama-cu.ac.jp (Y.I.); hasumi@yokohama-cu.ac.jp (H.H.); jikuya@yokohama-cu.ac.jp (R.J.); pjcbn002@yahoo.co.jp (A.H.); m7900828@yokohama-cu.ac.jp (M.Y.); jteran@yokohama-cu.ac.jp (J.-i.T.); hu0428@med.yokohama-cu.ac.jp (H.U.); 2 Department of Urology, Yokohama City University School of Medicine, Yokohama 236-0004, Japan; makiya@yokohama-cu.ac.jp; 3 Departments of Pathology & Laboratory Medicine and Urology, University of Rochester Medical Center, Rochester, NY 14642, USA; hiroshi_miyamoto@urmc.rochester.edu

**Keywords:** FGFR, urothelial carcinoma, erdafitinib, bladder cancer, muscle-invasive

## Abstract

**Introduction:** Bladder cancer is associated with a high recurrence rate, and outcomes for muscle-invasive and metastatic disease remain poor. New targeted therapies, such as the FGFR inhibitor erdafitinib, have been introduced, but the progression from non-muscle-invasive to muscle-invasive disease remains a major clinical challenge. **Methods:** In this study, we performed immunohistochemical staining for FGFR1-FGFR4 on surgical specimens from 192 cases of urothelial carcinoma. We also conducted various functional assays on human bladder cancer cell lines to assess protein/gene expression, cell proliferation, migration, invasion, and colony formation. **Results:** FGFR2 and FGFR3 expressions were found to be significantly down-regulated in high-grade (0.014) and muscle-invasive (0.002) tumors, respectively. Functionally, the FGFR inhibitor erdafitinib suppressed cell proliferation and migration, and FGFR3 silencing also markedly reduced proliferation, migration, invasion, and colony formation in cancer cell lines. **Conclusions:** The down-regulation of FGFR3 in muscle-invasive bladder cancer, coupled with the inhibitory effect of its inactivation on cell growth, suggests a significant role for FGFR3 in bladder cancer progression.

## Simple Summary

Muscle-invasive bladder cancer (MIBC) progression remains a major clinical challenge. This study found that FGFR3 expression is significantly down-regulated in tumors that have advanced to the muscle-invasive stage. To explore this, we conducted comprehensive functional assays. The FGFR inhibitor, erdafitinib, effectively suppressed cell growth in vitro. Crucially, specific silencing of FGFR3 consistently and dramatically reduced several aggressive cellular phenotypes, including proliferation, migration, invasion, and colony formation. These robust results strongly suggest that the down-regulation of FGFR3 is a crucial molecular event for bladder cancer progression. This mechanism positions FGFR3 as a highly promising and actionable therapeutic target for developing new treatments against aggressive MIBC.

## 1. Introduction

Bladder cancer is one of the most frequently diagnosed cancers, particularly among men [1]. Most bladder cancers are histologically urothelial carcinoma, while pure squamous cell carcinoma, adenocarcinoma, and neuroendocrine neoplasms such as small cell neuroendocrine carcinoma are occasionally seen. In addition, a subset of urothelial carcinomas exhibits histological variants, such as sarcomatoid, micropapillary, plasmacytoid, nested or microcystic, lymphoepithelioma-like, and giant cell features [2,3,4]. In Japan, the age-adjusted incidence rates of urothelial carcinoma by site were reported to be 7.2 per 100,000 population for bladder cancer, 2.68 per 100,000 population for renal pelvis and ureter cancer, and 0.06 per 100,000 population for urethral cancer [5].

Although a high recurrence rate is characteristic of bladder cancer, reported recurrence rates vary, ranging from 15 to 61% within 1 year after surgery and 31–78% within 5 years [6]. Additionally, in a study involving 426 patients with upper urinary tract cancer, postoperative recurrence within the bladder was seen in 18.3% of the cases within 1 year, 22.3% within 2 years, and 24.6% within 3 years, indicating that bladder cancer is not the only risk factor for recurrent urothelial cancer in the bladder. Because bladder tumors may not always be resected completely via transurethral endoscopic surgery following their diagnosis and some disparities in the surgical technique exist between surgeons or institutions, improvement in the quality of the initial surgical procedure is often challenging [7].

The treatment of urothelial carcinoma varies greatly depending on the tumor extent. The standard treatment for metastatic urothelial carcinoma is cisplatin-based combined chemotherapy. However, in recent years, immune–oncology agents as a second-line therapy and, starting in 2022, the anti-nectin-4 antibody–microtubule inhibitor complex, enfortumab vedotin, as a third-line therapy have also become treatment options (ref). Recently, in patients whose disease remained unchanged or progressed despite standard cisplatin therapy, a combination with the anti-PD-L1 antibody avelumab extended the progression-free survival (PFS) by 1.7 months and the overall survival (OS) by 7.1 months, compared with chemotherapy alone. Thus, the high recurrence rate of non-muscle-invasive bladder cancer (NMIBC), the risk of progression from NMIBC to muscle-invasive bladder cancer (MIBC) in high-risk groups, and low survival rates after progression to MIBC or metastatic disease, despite various treatment options currently available, are still unsatisfactory.

In February 2023, the results of the phase III JAVELIN Bladder 100 trial were reported with an observation period of more than two years and showed that enfortumab vedotin was effective in the first-line treatment of locally advanced or metastatic urothelial cancer [8]. In October 2023, the EV-302/KEYNOTE-A39 Phase III trial showed that the combination of enfortumab vedotin and pembrolizumab, an anti-PD-1 antibody, significantly improved the PFS by 6.2 months and the OS by 15.4 months, almost doubling both times compared with standard cisplatin-based chemotherapy. However, while these trials showed better oncologic outcomes than existing modalities, the survival rate remains low, and the results were still unsatisfactory [8,9].

FGFR3 alteration (mutation/fusion) is detected in over 70% of NMIBC, but this prevalence decreases sharply to approximately 10–20% in MIBC [A:24634132] [10]. This phenomenon is attributed to a molecular transition: NMIBC is largely dominated by the luminal subtype, which frequently harbors FGFR3 alterations and exhibits a high sensitivity to FGFR inhibitors [11,12,13]. Conversely, MIBC shows an increase in the basal subtype, which is less dependent on FGFR signaling and less sensitive to FGFR inhibitors [14]. This suggests that the reduction in FGFR3 expression is not coincidental but rather the result of a biological switching mechanism that occurs during the progression from NMIBC to MIBC [15]. In October 2023, a phase III THOR trial comparing FGFR inhibitors with chemotherapy in previously treated metastatic bladder cancer (NCT03390504) demonstrated that the primary endpoint, the median OS, was 7.8 months in the chemotherapy arm versus 12.1 months in the erdafitinib arm, with a 36% reduction in the risk of death in the erdafitinib arm [hazard ratio (HR): 0.64, 95% confidence interval: 0.47–0.88, *p* = 0.005]. In terms of safety, the rate of grade 3 or 4 treatment-related adverse events (TRAEs) was similar in the chemotherapy group (46.4%) and the erdafitinib group (45.9%), and the rate of death due to TRAEs was even lower in the erdafitinib group (5.4%) than in the chemotherapy group (0.7%) [16].

There are at least four FGFR isoforms, FGFR1-FGFR4. However, the functional role of FGFRs in bladder cancer progression remains to be further determined. In the present study, we assessed the expression of FGFRs in bladder cancer specimens and the impact of FGFR inhibition on the growth of bladder cancer cell lines.

## 2. Methods

### 2.1. Bladder Tissue Microarray (TMA) and Immunohistochemistry (IHC)

We obtained a set of bladder TMAs from US Biomax (Rockville, MD, USA). The TMA consisted of formalin-fixed paraffin-embedded tissues of 192 bladder tumors and 16 benign-appearing bladders from patients with tumors. All these tumors were classified according to the 2004 World Health Organization/International Society of Urological Pathology classification system for urothelial neoplasms.

IHC was performed on the sections (4 µm thick) from the bladder TMA. Slides were deparaffinized using xylene, rehydrated with graded ethanol, and subjected to heat-induced antigen retrieval by microwaving (2 min at high power, followed by 30 min at mid-low power) (Target Retrieval Solution pH9; Dako, Carpinteria, CA, USA). After blocking with 3% hydrogen peroxidase, samples were incubated overnight at 4 °C with a primary antibody against FGFR1 (clone 10F12E7, dilution 1:50; Proteintech, Rosemont, IL, USA), FGFR2 (clone 1G3, 5 μg/mL; Abnova, Taipei, Taiwan), FGFR3 (clone B-9, 1:50; Santa Cruz Biotechnology, Dallas, TX, USA), or FGFR4 (clone A-10, 1:50; Santa Cruz Biotechnology). The slides were then treated with a broad-spectrum secondary antibody (Histostain-SP Broad Spectrum Kit; Invitrogen, Grand Island, NY, USA) and washed with Envision FLEX Wash Buffer (Dako). After 3,3′-diaminobenzidine staining, the slides were counter-stained with hematoxylin, dehydrated with gradient ethanol and xylene, and then sealed. All slides were reviewed by a single pathologist (HM), who was blinded to the sample identities. The original pathology data, including the IHC slides and results, was obtained from USBioMAX. The German immunoreactive score was determined by multiplying the percentage of immunoreactive cells (0% = 0; 1–10% = 1; 11–50% = 2; 51–80% = 3; and 81–100% = 4) by the staining intensity (negative = 0; weak = 1; moderate = 2; and strong = 3). Immunohistochemical scores, ranging from 0 to 12, were classified as negative (0; 0–1), weakly positive (1+; 2–4), moderately positive (2+; 6–8), or strongly positive (3+; 9–12) for FGFR expression. By accounting for both the number of expressing cells (quantitative aspect) and the level of protein expression within them (qualitative aspect), the score provides a more biologically relevant and sensitive metric [D:9713356] [17].

### 2.2. Cell Culture and Chemicals

Human bladder urothelial carcinoma cell lines, UMUC3, TCC-sup, 5637, HTB3, and T24, were obtained from American Type Culture Collection (ATCC, Manassas, VA, USA). UMUC-3 and TCC-sup were maintained in Minimum Essential Medium (MEM; Thermo Fisher Scientific, Waltham, MA, USA), along with Non-Essential Amino Acid Solution (Thermo Fisher Scientific) and sodium pyruvate (Thermos Fischer Scientific). A total of 5637 cells were maintained in RPMI1640 (Thermos Fischer Scientific). HTB-3 cells were maintained in MEM. T24 cells were maintained in Dulbecco’s modified Eagle’s medium F12 (Thermo Fisher Scientific). All culture media were supplemented with 5% fetal bovine serum (FBS; Thermo Fisher Scientific), penicillin (100 units/mL), and streptomycin (100 units/mL), and the cells were cultured at 37 °C in a humidified atmosphere of 5% CO_2_. Small interfering RNA (siRNA) (sc-29314) was obtained from Santa Cruz Biotechnology, while erdafitinib (S8401) was from Selleck Biotech (Houston, TX, USA). The effective concentration range (IC_50_) for FGFR inhibition was determined based on the pharmacologic properties of erdafitinib and evaluation of dose–response relationships. According to Perera et al., in vitro assays reported an IC50 for erdafitinib ranging from 1.2 to 5.7 nM [E: 28341788] [18]. In our study, to accurately determine the dose–response in cell lines, we set concentrations at 1 nM and 10 nM to span this range. We also performed experiments at 0 nM as a control group.

### 2.3. Western Blotting

Protein extraction and Western blotting were performed. In brief, equal amounts of protein (10 µg) obtained from cell extracts were harvested for total protein analyses. The extracted protein was placed between two glass plates with polyacrylamide gel (e-PAGEL HR 10% 14 WELL; ATTO Corporation, Tokyo, Japan). Electroblotting was performed according to the standard protocol. Polyacrylamide gel was transcribed to a polyvinylidene fluoride membrane (Immune-Blot PVDF Membrane; Invitrogen, Waltham, MA, USA) before blocking. Specific antibody binding was detected using an FGFR1 (dilution 1:1000), FGFR2 (1:1000), FGFR3 (1:1000), FGFR4 (1:500), or β-actin (clone 13E5, dilution 1:10,000; Cell Signaling Technology, Danvers, MA, USA) antibody. Primary antibodies were washed 3 times (10 min each) with phosphate-buffered saline (PBS) (pH 7.4, 1000 mL of distilled water for one tablet; T9181; Takara Bio Inc., Kusatsu, Japan). A secondary mouse antibody (ECLTM Anti-mouse IgG Horseradish Peroxidase-linked whole antibody, NA931V; Cytiva, Marlborough, MA, USA) for FGFRsand a rabbit antibody (ECLTM Anti-rabbit IgG Horseradish Peroxidase-linked whole antibody: NA934V; Cytiva) for β-actin was used as a loading control. They were again washed 3 times with PBS for 10 min each. Finally, the membrane was stained with fluorescent solution (EzWestLumi plus; ATTO Corporation) and scanned using an infrared imaging system (LI-COR Odyssey; LI-COR, Lincoln, NE, USA).

### 2.4. Reverse Transcription (RT) and Real-Time PCR

Total RNA (0.5 μg) was isolated from cultured cells using PureLink^TM^ RNA Mini Kit (Invitrogen) in a total volume of 20 μL. Real-time quantitative PCR (qPCR) was then performed (StepOnePlus^TM^; Thermo Fisher Scientific) using iQTM Fast SYBR Green Master Mix (Thermo Fisher Scientific). The primer sets we purchased (all from Thermo Fisher Scientific) were as follows: Hs00241111_m1 (FGFR1, catalog No. 4331182); Hs01552918_m1 (FGFR2, catalog No. 4331182); Hs00179829_m1 (FGFR3, catalog No. 4331182); Hs01106910_g1 (FGFR4, catalog No. 4331182); and Hs01060665_g1(β-Actin, catalog No. 4331182).

### 2.5. Cell Proliferation Assay

The methyl thiazolyl diphenyl tetrazolium bromide (MTT) assay was used to assess cell viability. In brief, cells (0.5–1 × 10^3^/well) seeded in 96-well tissue culture plates were incubated with medium supplemented with FBS. The wells were divided equally for control (without erdafitinib) and erdafitinib treatment. After up to 48 h of culture, 10 μL of MTT stock solution (5 mg/mL; Sigma-Aldrich, St. Louis, MO, USA) was added to each well and incubated for 2 h at 37 °C. The absorbance was measured at a wavelength of 570 nm with background subtraction at 655 nm using a luminometer (xMark^TM^ Microplate Spectrophotometer; Bio-Rad, Hercules, CA, USA).

### 2.6. Cell Migration Assay

To assess cell migration, a wound healing assay was conducted. Cells were plated in 6-well plates at a density of 5 × 10^3^ cells/well. After achieving nearly 100% confluence, cells were scratched with a plastic tip to create a 1 mm wide clean wound area, incubated for 24 h in serum-free media, and fixed with methanol. Crystal violet (0.1% Crystal Violet Hydrate; Tokyo Chemical Industry, Tokyo, Japan) in PBS was used for staining. After photographing with an optical microscope, the area affected by cell migration was analyzed using the ImageJ software program v1.54j (National Institutes of Health, Bethesda, MD, USA).

### 2.7. Transwell Invasion Assay

Cell invasiveness was determined using Matrigel (BioCoat Matrigel Invasion Chamber; Corning, Corning, NY, USA). Erdafitinib (2.5 × 10^4^/well) or control cells in 500 µL of serum-free medium were placed in the upper chamber of the transwell, while 500 µL of serum-free medium was added to the lower chamber. After 48 h of incubation at 37 °C in a CO_2_ incubator, the invading cells were fixed, stained with 0.1% crystal violet, captured using a light microscope, and counted with the ImageJ software program v1.54j.

### 2.8. Clonogenic Assay

Cells seeded in 12-well plates (5 × 10^2^ cells/well) were allowed to grow until colonies in the control well were easily distinguishable, fixed with methanol, stained with crystal violet (0.1% Crystal Violet Hydrate) in PBS, and washed with distilled water. After photographing with an optical microscope, the number and area of colonies were quantified using the ImageJ software program v1.54j.

### 2.9. Statistical Analysis

All continuous variables are presented as the mean ± standard deviation. To assess the association between categorized variables, Fisher’s exact test or the χ^2^ test was applied. Nonparametric comparisons for two- and multi-group distributions were conducted using the Mann–Whitney U test and the Kruskal–Wallis test, respectively, to examine differences in variables with ordered distributions across dichotomous categories. The numerical data were compared using Student’s *t*-test. A *p* value of less than 0.05 was considered to be statistically significant.

## 3. Results

### 3.1. Expression of FGFRs in Bladder Cancer Specimens and Its Correlations with Histopathological Factors

**Figure 1:**
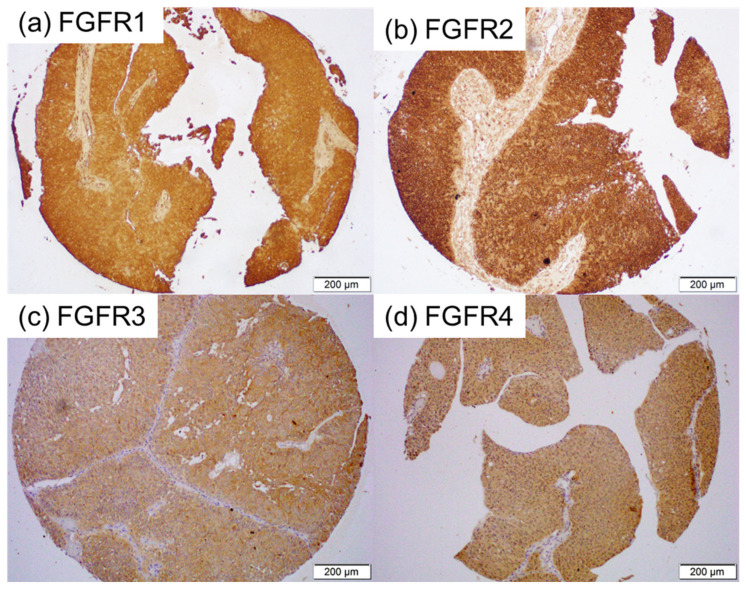
Immunohistochemistry in bladder cancer specimens. Immunostaining for FGFR1 (**a**), FGFR2 (**b**), FGFR3 (**c**), and FGFR4 (**d**) in the bladder TMA.

We immunohistochemically investigated the expression of FGFR1-FGFR4 in 192 bladder tumor specimens, along with 11 corresponding benign bladder tissues (excluding 5 cases showing no urothelium). A tumor was not present in some of the tissue cores in the TMA [for FGFR2 (n = 2) and FGFR4 (n = 1)]. Positive signals were detected predominantly in the cytoplasm of benign and malignant epithelial cells (Figure 1). The correlations of immunoreactivity with histopathologic features are summarized in Table 1, Table 2, Table 3 and Table 4.

FGFR1 was positive in all (2 with 1+, 4 with 2+, and 5 with 3+) of the 11 non-neoplastic urothelial tissues and all (19 with 1+, 66 with 2+, and 107 with 3+) of the 192 tumors [Table 1]. There were no significant differences in the levels of FGFR1 expression between normal tissue vs. tumors (*p* = 0.380), Grade 1–2 tumors vs. Grade 3 tumors (*p* = 0.259), or NMIBC vs. MIBC (*p* = 0.533).

FGFR2 was positive in all (1 with 1+, 4 with 2+, and 6 with 3+) of the 11 non-neoplastic urothelial tissues and all (30 with 1+, 79 with 2+, and 81 with 3+) of the 190 tumors [Table 2]. There were no significant differences in the levels of FGFR2 expression between the normal tissue vs. Cancer (*p* = 0.550) or NMIBC vs. MIBC (*p* = 0.152). However, strong FGFR2 expression was observed significantly more often in Grade 1–2 tumors than in Grade 3 tumors (*p* = 0.014).

FGFR3 was positive in 3 (27.3%; all 1+) of the 11 non-neoplastic urothelial tissues and 60 (31.3%; 42 with 1+, 17 with 2+, and 1 with 3+) of the 192 tumors [Table 3]. There were no significant differences in FGFR3 positivity between the normal tissue vs. Cancer (*p* = 0.782). However, marginal and significant differences in FGFR3 positivity were seen in Grade 1–2 tumors vs. Grade 3 tumors (*p* = 0.093) and NMIBC vs. MIBC (*p* = 0.002), respectively.

FGFR4 was positive in 6 (54.5%; all 1+) of the 11 non-neoplastic urothelial tissues and 84 (42.4%; 77 with 1+ and 7 with 2+) of the 191 tumors [Table 4]. There were no significant differences in FGFR4 positivity between the normal tissue vs. Cancer (*p* = 0.734), Grade 1–2 tumors vs. Grade 3 tumors (*p* = 0.712), or NMIBC vs. MIBC (*p* = 0.131).

### 3.2. Expression of FGFRs in Bladder Cancer Cell Lines

**Figure 2:**
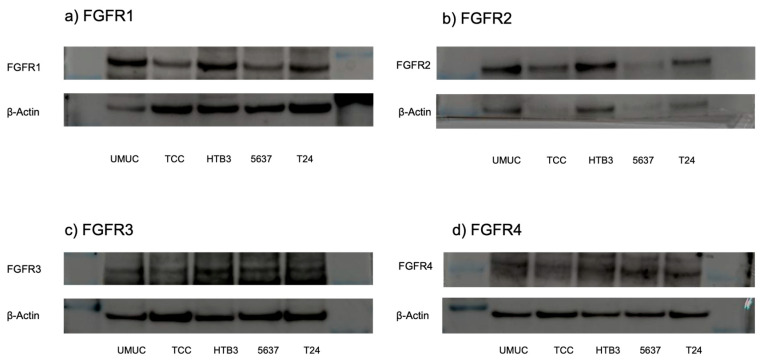
Expression of FGFR proteins in bladder cancer cell lines. Western blotting of FGFR1 ((**a**); 91 kDa), FGFR2 ((**b**); 37 kDa), FGFR3 ((**c**); 125–135 kDa), and FGFR4 ((**d**); 95–125 kDa) in UMUC3, TCC-sup, HTB3, 5637, and T24 cells. β-Actin (45 kDa) served as an internal control. The uncropped blots are shown in Figure S1 in Supplementary Materials.

**Figure 3:**
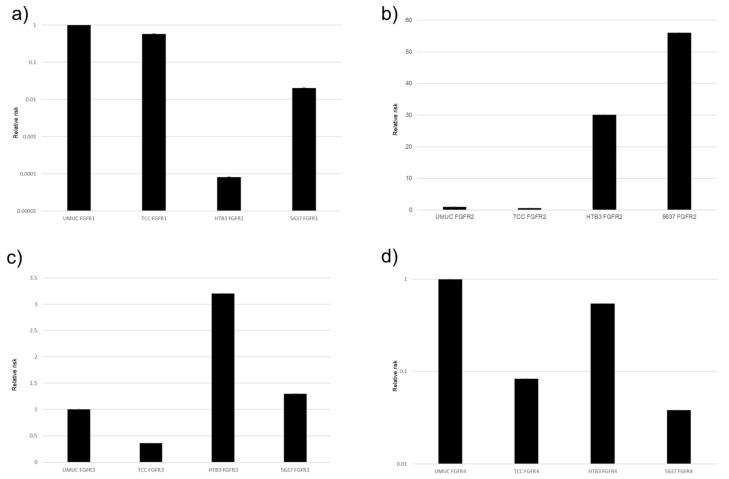
Expression of *FGFR* genes in bladder cancer cell lines. qPCR for FGFR1 (**a**), FGFR2 (**b**), FGFR3 (**c**), and FGFR4 (**d**) in UMUC3, TCC-sup, HTB3, and 5637 cells. Expression of each specific gene normalized to that of β-actin relative to FGFR represents the mean (+SD) from at least three independent experiments.

The protein and gene expression of FGFR1-FGFR4 were examined in five bladder urothelial cancer cell lines, using Western blotting (Figure 2) and real-time qPCRs (Figure 3), respectively. All these cell lines were confirmed to express FGFR1–FGFR4.

### 3.3. Effect of Erdafitinib on Bladder Cancer Cell Growth

**Figure 4:**
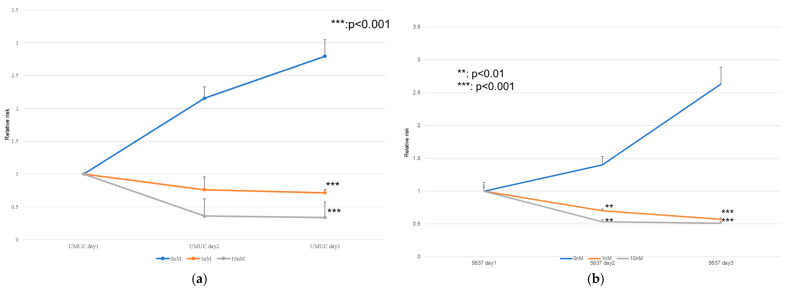
Effect of erdafitinib on the proliferation of bladder cancer cell lines. UMUC3 (**a**) and 5637 (**b**) cells were cultured for 1–3 days with 0–10 nM of erdafitinib, and cell viability was assayed with MTT. Growth induction is presented. Each value relative to the cell number of each line at day 1 represents the mean (+SD) from at least three independent experiments. ** *p* < 0.01; *** *p* < 0.001 (vs. mock treatment).

**Figure 5:**
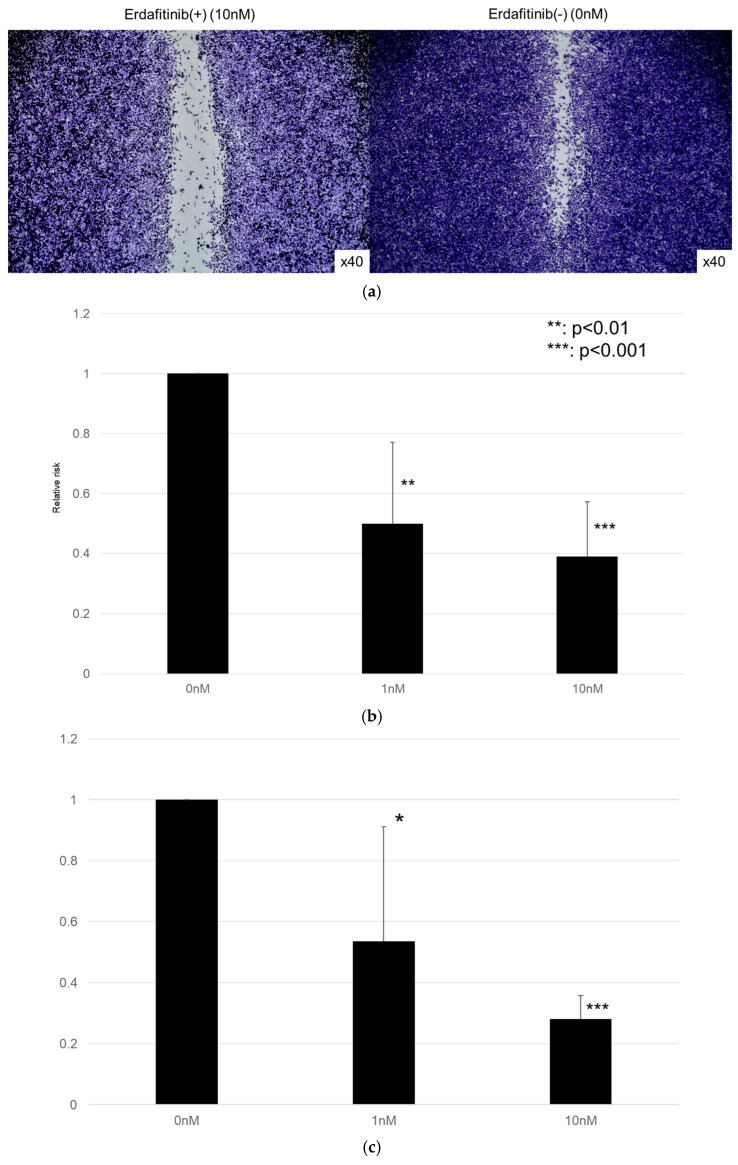
Effect of erdafitinib on the migration of bladder cancer cell lines. UMUC3 (**a**,**b**) and 5637 (**c**) cells grown to confluence on 6-well tissue culture plates were gently scraped, and the wound closure area was measured 24 h after treatment with 0–10 nM of erdafitinib. Each value relative to the migration of mock-treated cells represents the mean (+SD) from three or more independent experiments. * *p* < 0.05; ** *p* < 0.01; and *** *p* < 0.001 (vs. mock treatment).

**Figure 6:**
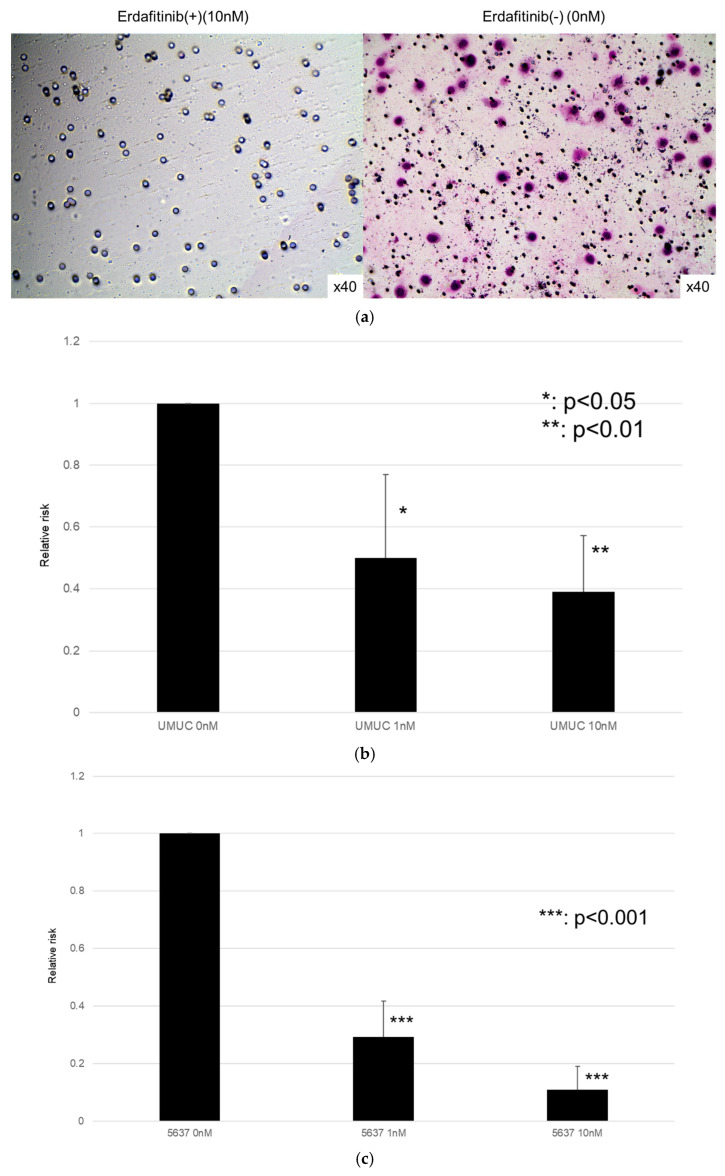
Effects of erdafitinib on cell invasion in bladder cancer cell lines. UMUC3 (**a**,**b**) and 5637 (**c**) cells were cultured in Matrigel-coated transwell chambers in the presence of 0–10 nM erdafitinib for 48 h, and the number of infiltrating cells in the lower chambers was counted in five random fields under a light microscope (10× objective). Each value relative to the number of mock-treated cells represents the mean (+SD) from three independent experiments. * *p* < 0.05; ** *p* < 0.01; and *** *p* < 0.001 (vs. mock treatment).

The effects of erdafitinib on the cell growth were analyzed via an MTT assay (Figure 4), wound healing assay (Figure 5), and transwell invasion assay (Figure 6). In the MTT assay, erdafitinib significantly reduced the cell proliferation of UMUC3 (Figure 4a) and 5637 (Figure 4b) in a dose-dependent manner. In the wound healing assay, erdafitinib at 1 or 10 nM significantly inhibited the migration of UMUC3 (Figure 5a,b) and 5637 (Figure 5c) cells. Similarly, in the transwell invasion assay, erdafitinib at 1 or 10 nM significantly inhibited the invasion of UMUC3 (Figure 6a,b) and 5637 (Figure 6c) cells.

### 3.4. Effect of FGFR3 Silencing

**Figure 7:**
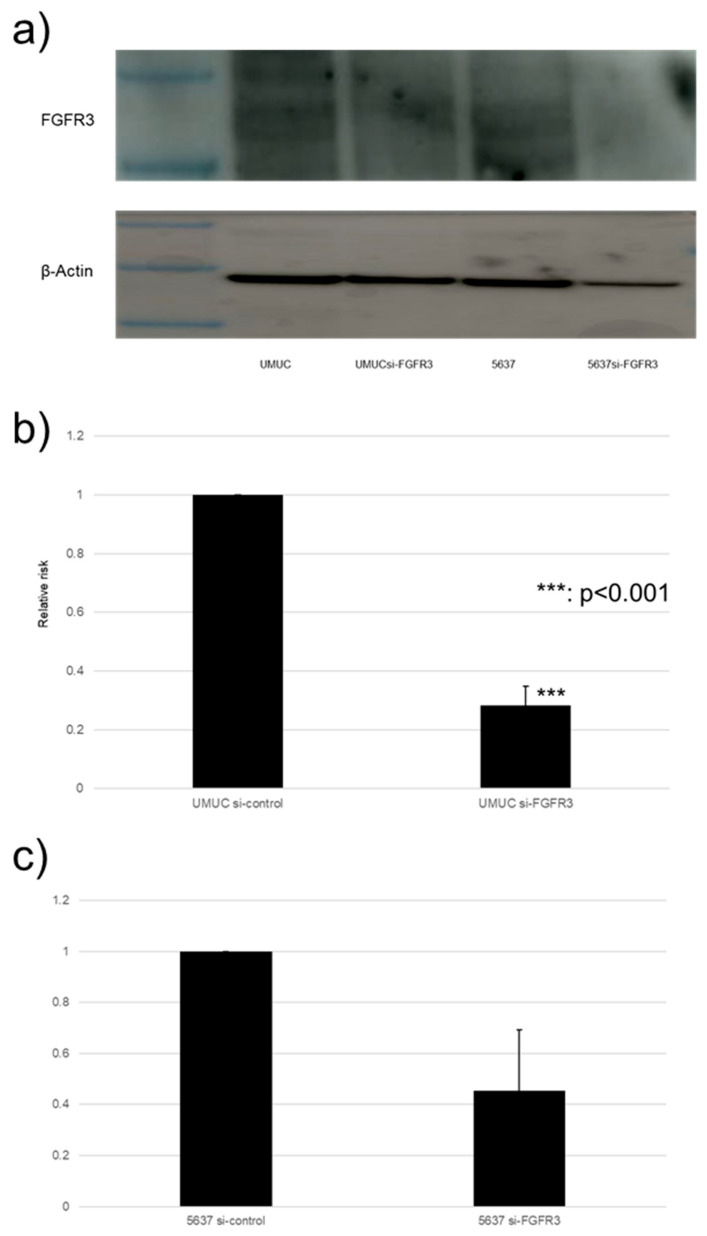
FGFR3 silencing in bladder cancer cell lines. (**a**) Western blotting of FGFR3 (125–135 kDa) in UMUC3 and 5637 cells expressing control-siRNA or FGFR3-siRNA. β-Actin (45 kDa) served as an internal control. (**b**,**c**) qPCR of *FGFR3* in UMUC3 and 5637 cells expressing control-siRNA or FGFR3-siRNA. Each value relative to mock-treated transcript levels in each cell line represents the mean (+SD) from at least three independent experiments. *** *p* < 0.001 (vs. control-siRNA). The uncropped blots are shown in Figure S1.

We focused on assessing the functional role of FGFR3 in the growth of bladder cancer cells. FGFR3 was silenced in UMUC3 and 5637 cells via the transfection of its siRNA. The Western blotting and qPCR (Figure 7) confirmed the reduced expression of FGFR3 in cells expressing FGFR3-siRNA, compared with those expressing the control-siRNA.

**Figure 8:**
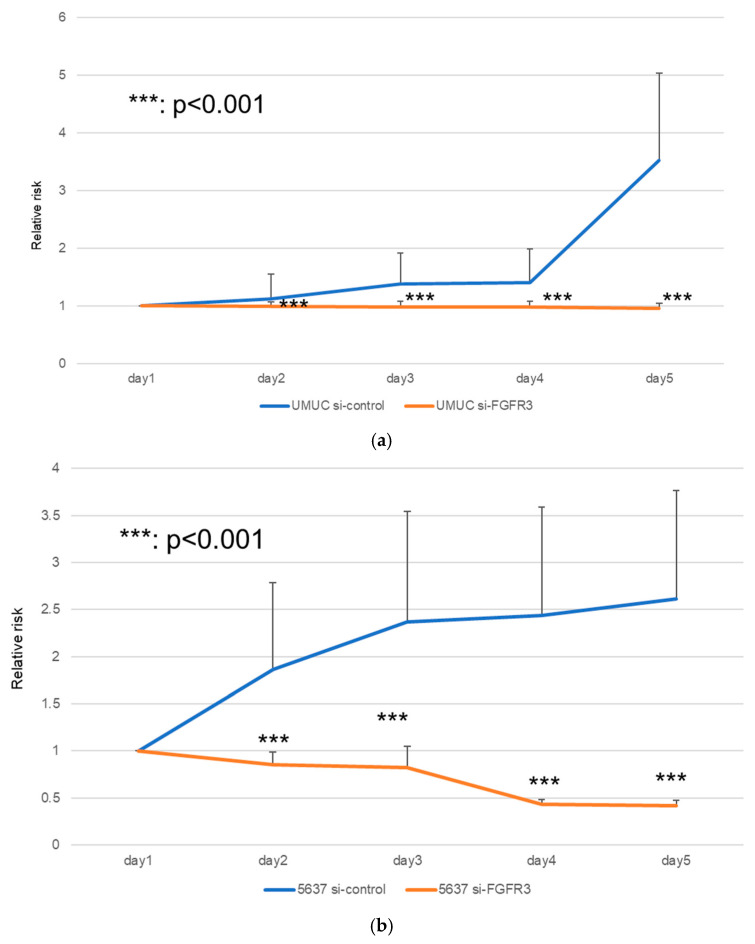
Effects of FGFR3 silencing on the proliferation of bladder cancer cell lines. UMUC3 (**a**) and 5637 (**b**) cells expressing control-siRNA or FGFR3-siRNA were cultured for 1–5 days, and cell viability was assayed by MTT. Each value relative to the cell number of each line at day 1 represents the mean (+SD) from at least three independent experiments. *** *p* < 0.001 (vs. control-siRNA).

**Figure 9:**
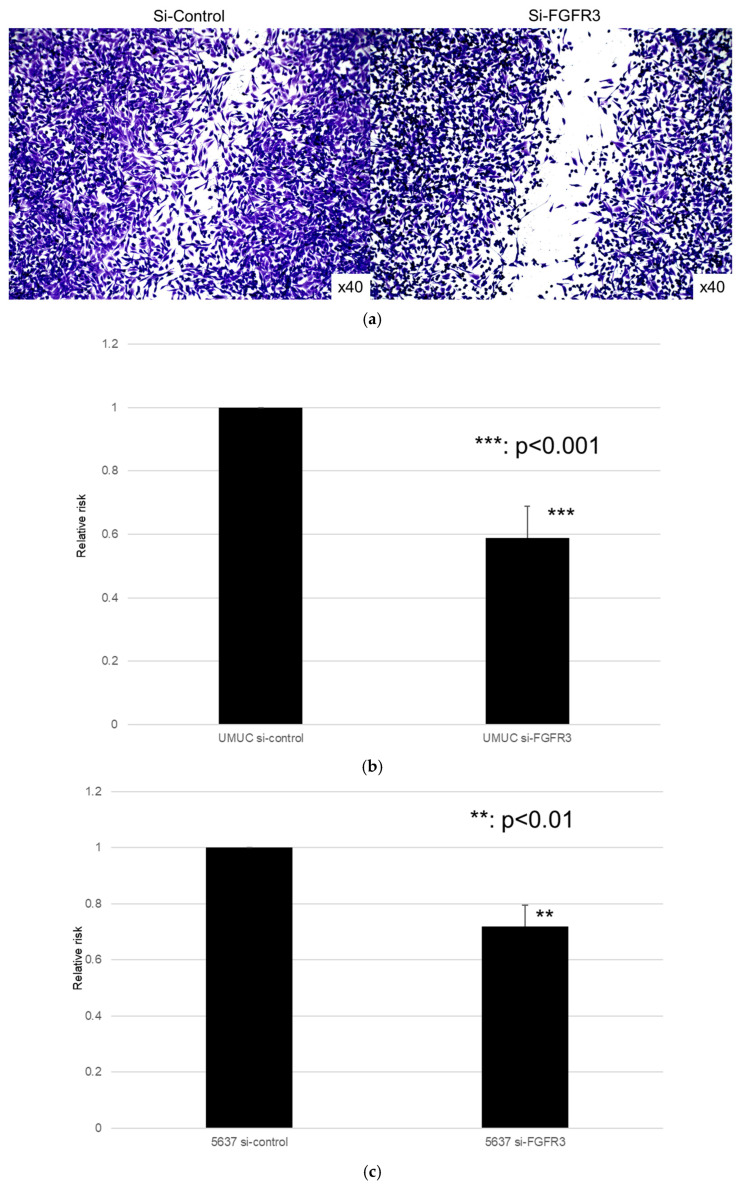
Effect of FGFR3 silencing on the migration of bladder cancer cell lines. UMUC3 (**a**,**b**) and 5637 (**c**) cells expressing control-siRNA or FGFR3-siRNA grown to confluence on 6-well tissue culture plates were gently scraped, and the wound closure area was measured after 24 h. Each value relative to the migration of control cells represents the mean (+SD) from three or more independent experiments. ** *p* < 0.01, *** *p* < 0.001 (vs. control-siRNA).

**Figure 10:**
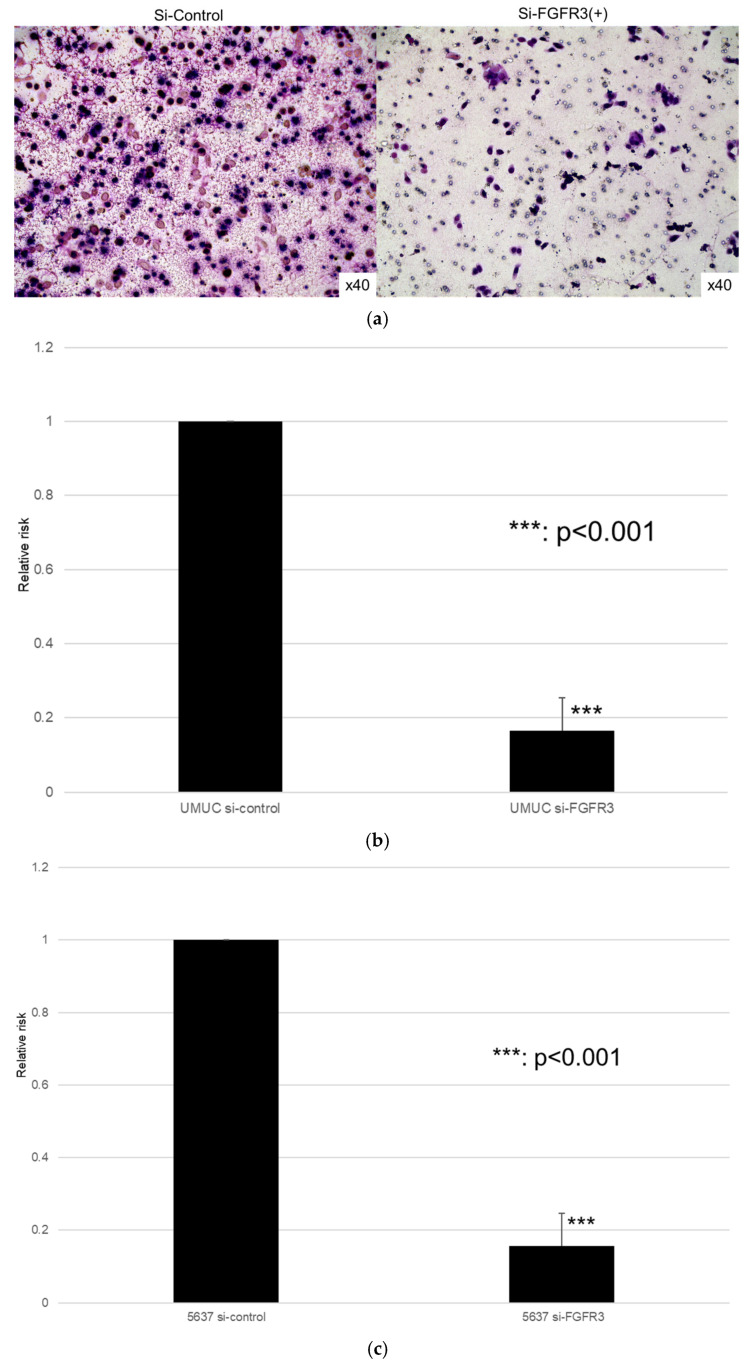
Effects of FGFR3 silencing on the invasion of bladder cancer cell lines. UMUC3 (**a**,**b**) and 5637 (**c**) cells expressing control-siRNA or FGFR3-siRNA were cultured in Matrigel-coated transwell chambers for 48 h, and the number of infiltrating cells in the lower chambers was counted in five random fields under a light microscope (10× objective). Each value relative to the invasion of control cells represents the mean (+SD) from three independent experiments. *** *p* < 0.001 (vs. control-siRNA).

**Figure 11:**
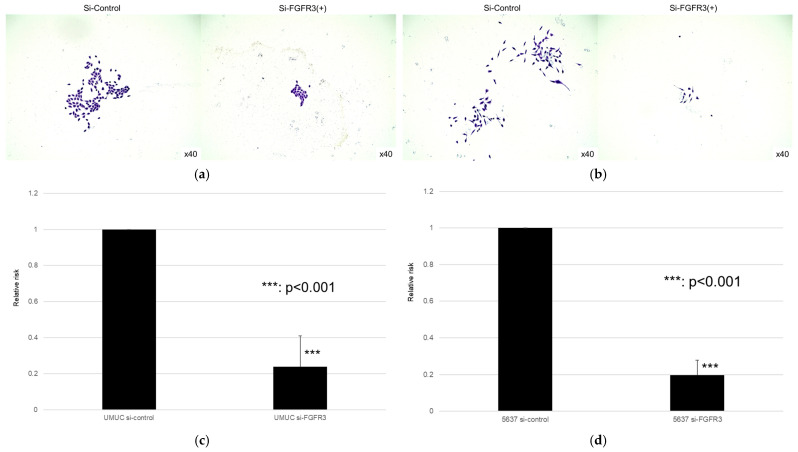
Effect of FGFR3 silencing on the colony formation in bladder cancer cell lines. UMUC3 (**a**,**b**) and 5637 (**c,d**) cells expressing control-siRNA or FGFR3-siRNA were cultured, and the number of the colonies was counted. Each value relative to the number of control colonies represents the mean (+SD) from at least three independent experiments. *** *p* < 0.001 (vs. control-siRNA).

We next assessed the impact of FGFR3 silencing on the growth of UMUC3 and 5637 cells, using an MTT assay (Figure 8), a wound healing assay (Figure 9), an invasion assay (Figure 10), and a clonogenic assay (Figure 11). As expected, FGFR3 silencing resulted in significant inhibition in the cell proliferation, cell migration, cell invasion, and colony formation of both lines.

## 4. Discussion

The early stage of bladder cancer is called NMIBC. And when it has progressed, it infiltrates into the muscle layer, which is called MIBC. The standard treatment for MIBC is chemotherapy and radical cystectomy, and even if these are performed, the 5-year survival rate is 40–60% at relapse [19]. Treatment options for NMIBC have not changed significantly, only with bladder injections called TURBT and injections into bladder such as BCG and THP after TURBT. Even when treated with endoscopic resection and bladder injections, reported recurrence rates range from 15% to 61% within 1 year after surgery and 31% to 78% within 5 years. And among that, 15–20% of these recurrences become MIBC [6]. This is a condition for which no probabilistic treatment exists. Though some clinically important factors have been raised, useful factors linked to therapy have yet to be identified [F:40557582], [G:40647487] [20,21]. FGFR3 inhibitors were approved in Japan in December 2024 for the treatment of invasive cancer in the muscle layer that cannot be curatively resected. However, our results showed that FGFR3 expression was higher in NMIBC than in MIBC. In former studies, Akanksha et al. reported that FGFR3 expression in MIBC was positive in 18.2% of cases, compared to 66.7% in high-grade and 82.6% in low-grade NMIBC; overall FGFR3 expression in NMIBC was found in 78.1% of cases, showing a significant difference (*p* < 0.05) [H:31528172] [22]. We also found an elevated FGFR3 expression in NMIBC (*p* < 0.05). Furthermore, although there was no significant difference (*p* = 0.093), the positive rate of FGFR3 in low-grade lesions (G1,2) was 35.3%, while that in high-grade lesions (G3) was 20.8%. Although there are few reports of studies using immunostaining for FGFR3 according to malignancy, Chang et al. reported that with FGFR3 immunostaining in multiple myeloma, not all the high-grade multiple myeloma showed the high expression level of FGFR3. And also, they mentioned that there was no relevance between high-grade multiple myeloma and the high expression level of FGFR3 [23].

In addition, although FGFR2 immunostaining in cholangiocarcinoma has been reported, the sensitivity of FGFR immunostaining remains low, with a sensitivity of 57.1% and a specificity of 97.7% according to Uson et al. Since NMIBC accounts for approximately 70–80% of bladder cancers, the evaluation of FGFR3 in MIBC may be inadequate [24]. However, in our study, the positivity rate is high in NMIBC. And there is the fact that among NMIBCs, approximately 20–25% of the high-risk group progresses to MIBC, and the 5-year survival rate is 40–60% at relapse. Given these facts, if a patient is determined to be FGFR3-positive at the time of NMIBC, there is room to consider the early introduction of erdafitinib, given the high specificity of FGFR3 immunostaining.

In the PCR, the descending order of expression levels was FGFR1, FGFR2, FGFR4, and FGFR3 in UMUC, and similar results were observed with the Western blotting. Next, for about 5637 cells with the PCR, the descending order of expression levels was FGFR1, FGFR2, FGFR4, and FGFR3. With the Western blotting, FGFR1 and FGFR4 were higher than FGFR2 and FGFR3. However, when expressions of FGFR1 to FGFR4 were compared with those of other cell lines (TCC, HTB3, and T24), FGFR3 was found to be significantly less strongly expressed than the other three FGFRs. 

Erdafitinib is the main inhibitor of FGFR3, and our results do not indicate that FGFR3 itself is not expressed but rather that FGFR1 and 2 are more highly expressed than FGFR3. Tomlinson et al. reported FGFR3 mutations in 42% of patients with urothelial carcinoma. They also found an overexpression of FGFR3 in 85% of these patients, suggesting an association between mutations and increased expression [25]. The expression of FGFR3 is high in urothelial carcinoma, and it has been concluded that FGFR3 is a predictive marker for urothelial carcinoma [26,27], especially in a Japanese report by Ikeda et al. [28], which differs from the present findings. However, in the study by Sternberg et al. involving only advanced and metastatic urothelial carcinoma, patients with an overexpression of FGFR1 or FGFR3 were divided into two groups: one receiving the FGFR1–4 inhibitor logaratinib and one receiving conventional cisplatin-based chemotherapy. The overall response rate was 20.7% in the logaratinib group and 19.3% in the chemotherapy group, and the median overall survival was 8.3 and 9.8 months, respectively (*p* = 0.67), findings that were comparable to the chemotherapy group. They also found that 69% of patients had high FGFR1 expression, suggesting that FGFR3 mutations associated with FGFR1 and FGFR3 overexpression may be a better predictor of a logaratinib response [29]. Kohler et al. reported that FGFR1 is also associated with squamous cell carcinoma of the lung and that the higher the copy number of the gene, the higher the expression of FGFR1. De Souza et al. reported that FGFR2 and FGFR3 mutations and fusions are prognostic biomarkers of urothelial carcinoma [30]. Mahmoud et al. reported that 60 invasive urothelial carcinomas were immunostained for FGFR2 and FGFR3 and found that the higher the grade, the higher the FGFR2 positivity rate (*p* = 0.044) and the more advanced the disease, the higher the FGFR2 positivity rate (*p* = 0.048) [31]. Therefore, the present results show that FGFR1 and FGFR22 are more highly expressed than FGFR3, which is consistent with previous reports.

The MTT assay performed on UMUC3 and 5637 showed a decrease in the cell count in the erdafitinib-treated group over the course of the day, whereas the cell count increased in the untreated group. Erdafitinib-treated patients also showed a volume-dependent decrease in cell count. The invasion assay showed the highest cell invasiveness in the control group, whereas the erdafitinib-treated group showed a volume-dependent decrease in cell invasiveness, and the migration assay showed similar results. The erdafitinib treatment resulted in a volume-dependent reduction in cell migration compared to the control group, which Kim et al. have also shown to be associated with the FGFR signaling pathway in breast cancer cells (BT-474). Therefore, when 10 nM of erdafitinib was administered to BT-474, a decrease in cell viability of less than 25% was observed in the MTT assay [32]. In addition, Jin et al. examined the effect of erdafitinib on bladder cancer cell lines (T24 and UMUC6) and reported a significant inhibition of cell migration and invasion by erdafitinib in invasion and migration assays [33]. We also reported that the expression of FGFR1 and FGFR4 was higher than that of FGFR2 and FGFR3 in UMUC and 5637 cells, but since erdafitinib is mainly an inhibitor of FGFR3, we performed an MTT assay on UMUC and 5637 cells with FGFR3 knockdown as well as invasion, migration, and colony assays. In the MTT assay, the number of cells in the control group increased over time, whereas the number of cells in the knockdown group decreased; in the invasion assay, cell invasion was suppressed in the control group, and in the migration assay, cell migration was similarly suppressed by the FGFR 3 knockdown, while the FGFR3 knockdown suppressed cell formation in the colony assay. Kim et al. reported that FGFR1 and 4 were highly expressed among FGFR1–4 and that the knockdown of FGFR1 and FGFR4 in the MTT assay showed a slight but significant decrease in cell viability [32]. Cheng et al. performed FGFR1 and FGFR3 knockdowns in bladder cancer cell lines (UMUC14 and RT4 cells). The MTT assay showed that the FGFR3 knockdown in RT4 and UM-UC14 cells was 50% and 80% more efficient than the FGFR1 and FGFR3 knockdown, respectively. Western blotting also showed that the FGFR3 knockdown resulted in a lower expression than in the control group. In contrast, the knockdown of FGFR1 had no significant effect on proliferation, indicating that FGFR3 plays a more important role than FGFR1 in promoting urothelial carcinoma growth [34]. Therefore, although the expression of FGFR1 was higher than that of FGFR3 in the present study, the authors reaffirmed the important role of FGFR3 in the development and progression of bladder cancer, and our results are consistent with those of previous reports.

Although FGFR3 inhibitors are currently used only after second-line therapy, it has been pointed out that among NMIBCs, approximately 20–25% of high-risk patients progress to MIBC and that more than half of NMIBC patients with high-risk disease will relapse [19]. Given the importance of inhibiting the progression of NMIBC to MIBC as an unmet need and the fact that FGFR3 is more highly expressed in NMIBC than in MIBC, the timing of the identification of FGFR3 mutations and the timing of therapeutic interventions using erdafitinib are also future issues to address. Erdafitinib is the only FGFR inhibitor available as a treatment option for bladder cancer. Our results suggest that other FGFR inhibitors may also be promising candidates for future therapeutic strategies.

This study is subject to several limitations. First, the non-tumor sites were obtained from cancer patients, so it was not possible to compare the normal mucosa of non-cancer patients with the tumor sites of cancer patients. In addition, the tissue samples were purchased, so details such as the treatment history of the patients are unknown. The absence of this information limited the ability to evaluate the significance of specific molecular markers as independent prognostic predictors. Consequently, a strict comparison between tumor-related changes and true physiological states was difficult, necessitating caution in interpreting the results. Second, it was not possible to elucidate which factors of FGFR are involved in tumor growth. And the detailed mechanisms governing therapeutic responses remain unknown, but we hypothesize signaling pathways (p-ERK/p-AKT/JAK–STAT) and the Epithelial–Mesenchymal Transition (EMT) will provide predictive clues in our future study [I:31987043], [J:19588205], [K:36675289] [35,36,37]. However, we were able to analyze the function of FGFR at the cellular level in bladder cancer, which is now being applied clinically, so additional studies are needed in the future. Third, this study performed an investigation in UMUC-3 and 5637 cell lines. The UMUC3 cell line lacks known FGFR1–4 mutations or fusion genes and functions as a standard muscle-invasive bladder cancer (MIBC) model exhibiting a low dependence on FGFR signaling. The 5637 cell line is known as a strain exhibiting an overexpression of wild-type FGFR3, although it lacks gain-of-function mutations in FGFR3 (FGFR3-TACC3 fusion gene or other activating mutations). By focusing on these two lines, we believed we could cover the spectrum of therapeutic responses, encompassing both cells dependent on FGFR3 gene mutations or fusions and cells exhibiting wild-type FGFR overexpression or non-dependence. Furthermore, both cell lines represent distinct clinical subtypes of bladder cancer. These two lines represent the major molecular subtypes of bladder cancer: the luminal subtype (5637) [L:31563503], [M:24520177], which is the primary subtype of non-muscle-invasive bladder cancer (NMIBC), and the basal cell subtype (UMUC3) [N:30038946], [O:25997541], representing both the major molecular subtypes of non-muscle-invasive bladder cancer (NMIBC) and muscle-invasive bladder cancer (MIBC), respectively. We believed that this enhances the clinical external validity and used these cell lines [P:24476821] [38,39,40]. Further study is needed to confirm the results in the other cell lines.

## 5. Conclusions

In conclusion, a functional analysis of FGFR3 in bladder cancer and the tumor suppressive effect of FGFR inhibitors were confirmed in various urothelial carcinoma cell lines. FGFR3 was found more frequently in bladder cancer and is thought to be involved in tumor growth. However, in contrast to other cancer types, we found that the expression of FGFR3 is not necessarily stronger in higher grades of cancer. Our findings establish only a preclinical rationale and do not evaluate patient outcomes, thus requiring cautious interpretation regarding early clinical adoption. Further prospective studies are essential to validate these mechanisms as prognostic or predictive biomarkers for therapy.

## Figures and Tables

**Table 1:** FGFR1 expression.

Variables	n	Immunohistochemical Expression				*p* Value
		0+	1+	2+	3+	0+ 1+ vs. 2+ 3+
Normal	11	0 (0.0%)	2 (18.2%)	4 (36.4%)	5 (45.5%)	0.38
Cancer	192	0 (0.0%)	19 (9.9%)	66 (34.4%)	107 (55.7%)	
NMIBC	52	0 (0.0%)	4 (7.7%)	22 (42.3%)	26 (50.0%)	0.533
MIBC	140	0 (0.0%)	15 (10.7%)	44 (31.4%)	81 (57.9%)	
Male	152	0 (0.0%)	18 (11.8%)	51 (33.6%)	83 (54.6%)	0.132
Female	40	0 (0.0%)	1 (2.5%)	15 (37.5%)	24 (60.0%)	
G1	26	0 (0.0%)	4 (15.4%)	6 (23.1%)	16 (61.5%)	0.259
G2	110	0 (0.0%)	11 (10.0%)	40 (36.4%)	59 (53.6%)	(G1,2 vs. G3)
G3	53	0 (0.0%)	3 (5.7%)	19 (35.8%)	31 (58.5%)	
Not available	3					

NMIBC: non-muscle-invasive bladder cancer, MIBC: muscle-invasive bladder cancer.

**Table 2:** FGFR2 expression.

Variables	n	Immunohistochemical Expression				*p* Value
		0+	1+	2+	3+	0+ 1+ vs. 2+ 3+
Normal	11	0 (0.0%)	1 (9.1%)	4 (36.4%)	6 (54.5%)	0.55
Cancer	190	0 (0.0%)	30 (15.8%)	79 (41.6%)	81 (42.6%)	
NMIBC	52	0 (0.0%)	5 (9.6%)	21 (40.4%)	26 (50.0%)	0.152
MIBC	138	0 (0.0%)	25 (18.1%)	58 (42.0%)	55 (39.9%)	
Male	150	0 (0.0%)	27 (18.0%)	68 (45.3%)	55 (36.7%)	0.143
Female	40	0 (0.0%)	3 (7.5%)	11 (27.5%)	26 (65.0%)	
G1	26	0 (0.0%)	3 (11.5%)	8 (30.8%)	15 (57.7%)	0.014
G2	108	0 (0.0%)	11 (10.2%)	49 (45.4%)	48 (44.4%)	(G1,2 vs. G3)
G3	53	0 (0.0%)	13 (24.5%)	22 (41.5%)	18 (34.0%)	
Not available	5					

NMIBC: non-muscle-invasive bladder cancer, MIBC: muscle-invasive bladder cancer.

**Table 3:** FGFR3 expression.

Variables	n	Immunohistochemical Expression				*p* Value
		0+	1+	2+	3+	0+ vs. 1+ 2+ 3+
Normal	11	8 (72.7%)	3 (27.3%)	0 (0.0%)	0 (0.0%)	0.782
Cancer	192	132 (68.8%)	42 (21.9%)	17 (8.9%)	1 (0.5%)	
NMIBC	52	27 (51.9%)	16 (30.8%)	9 (17.3%)	0 (0.0%)	0.002
MIBC	140	105 (75.0%)	26 (18.6%)	8 (5.73%)	1 (0.7%)	
Male	152	107 (70.4%)	33 (21.7%)	11 (7.2%)	1 (0.7%)	0.344
Female	40	25 (62.5%)	9 (22.5%)	6 (15.0%)	0 (0.0%)	
G1	26	22 (84.6%)	3 (11.5%)	1 (3.8%)	0 (0.0%)	0.093
G2	110	66 (60.0%)	30 (27.3%)	14 (12.7%)	0 (0.0%)	(G1,2 vs. G3)
G3	53	41 (77.4%)	9 (17.0%)	2 (3.8%)	1 (1.9%)	
Not available	3					

NMIBC: non-muscle-invasive bladder cancer, MIBC: muscle-invasive bladder cancer.

**Table 4:** FGFR4 expression.

Variables	n	Immunohistochemical Expression				*p* Value
		0+	1+	2+	3+	0+ vs. 1+ 2+ 3+
Normal	11	5 (45.5%)	6 (54.5%)	0 (0.0%)	0 (0.0%)	0.734
Cancer	191	107 (56.0%)	77 (40.3%)	7 (3.7%)	0 (0.0%)	
NMIBC	52	28 (53.8%)	22 (42.3%)	2 (3.8%)	0 (0.0%)	0.131
MIBC	139	79 (56.8%)	55 (39.6%)	5 (3.6%)	0 (0.0%)	
Male	151	83 (55.0%)	63 (41.7%)	5 (3.3%)	0 (0.0%)	0.596
Female	40	24 (60.0%)	14 (35.0%)	2 (5.0%)	0 (0.0%)	
G1	26	21 (80.8%)	5 (19.2%)	0 (0.0%)	0 (0.0%)	0.712
G2	109	56 (51.4%)	47 (43.1%)	6 (5.5%)	0 (0.0%)	(G1,2 vs. G3)
G3	53	27 (50.9%)	25 (47.2%)	1 (1.9%)	0 (0.0%)	
Not available	4					

NMIBC: non-muscle-invasive bladder cancer, MIBC: muscle-invasive bladder cancer.

## Data Availability

We attached raw data as Supplementary files of this manuscript. Due to ethical restrictions, the raw data underlying this study are available upon request from the corresponding author.

## Supplementary Materials

The following supporting information can be downloaded at: https://www.mdpi.com/article/10.3390/cancers17213588/s1.
