# Supplementary material for: The Role of FGFR3 in the Progression of Bladder Cancer
Source: Cancers (Basel). 2025 Nov 6;17(21):3588. doi: 10.3390/cancers17213588 (PMC12610005; doi:10.3390/cancers17213588)
Supplement: Supplementary file 1 [file cancers-17-03588-s001.zip › Figure S10.pdf]

	UMUC si-c	UMUC si-FGFR3
mean	1	0.240268
STDEV	0	0.169237

	5637 si-co	5637 si-FGFR3
mean	1	0.195858
STDEV	0	0.083098
