# Supplementary material for: The Role of FGFR3 in the Progression of Bladder Cancer
Source: Cancers (Basel). 2025 Nov 6;17(21):3588. doi: 10.3390/cancers17213588 (PMC12610005; doi:10.3390/cancers17213588)
Supplement: Supplementary file 1 [file cancers-17-03588-s001.zip › Figure S2.pdf]

| | |
|---|---|
| UMUC FGFR1 | 1 |
| TCC FGFR1 | 0.580206 |
| HTB3 FGFR1 | 8.14E-05 |
| 5637 FGFR1 | 0.02024 |

| | | | |
|---|---|---|---|
| UMUC FGFR1 | 0.024022 | 0.024022 | 1 |
| TCC FGFR1 | 0.013938 | 0.024022 | 0.580206 |
| HTB3 FGFR1 | 1.95E-06 | 0.024022 | 8.14E-05 |
| 5637 FGFR1 | 0.000486 | 0.024022 | 0.02024 |

| | | |
|---|---|---|
| UMUC FGFR4 | 0.000169 | 0.000169 |
| TCC FGFR4 | 1.41E-05 | 0.000169 |
| HTB3 FGFR4 | 9.16E-05 | 0.000169 |
| 5637 FGFR4 | 6.4E-06 | 0.000169 |

| | |
|---|---|
| UMUC FGF | 1 |
| TCC FGFR4 | 0.083265 |
| HTB3 FGFR | 0.542411 |
| 5637 FGFR | 0.037943 |
