# Supplementary material for: The Role of FGFR3 in the Progression of Bladder Cancer
Source: Cancers (Basel). 2025 Nov 6;17(21):3588. doi: 10.3390/cancers17213588 (PMC12610005; doi:10.3390/cancers17213588)
Supplement: Supplementary file 1 [file cancers-17-03588-s001.zip › Figure S3.pdf]

	UMUC day	UMUC day	UMUC day3
0nM	1	2.1538	2.796
1nM	1	0.76	0.7132
10nM	1	0.359	0.337

	5637 day1	5637 day2	5637 day3
0nM	1	1.402247	2.631461
1nM	1	0.701537	0.572527
10nM	1	0.532336	0.510422
