# Supplementary material for: The Role of FGFR3 in the Progression of Bladder Cancer
Source: Cancers (Basel). 2025 Nov 6;17(21):3588. doi: 10.3390/cancers17213588 (PMC12610005; doi:10.3390/cancers17213588)
Supplement: Supplementary file 1 [file cancers-17-03588-s001.zip › Figure S4.pdf]

UMUC 0nM	UMUC 1nM	UMUC 10nM
1	0.49888	0.390344

5637	0nM	1nM	10nM
mean	1	0.535657	0.280506
