# Supplementary material for: The Role of FGFR3 in the Progression of Bladder Cancer
Source: Cancers (Basel). 2025 Nov 6;17(21):3588. doi: 10.3390/cancers17213588 (PMC12610005; doi:10.3390/cancers17213588)
Supplement: Supplementary file 1 [file cancers-17-03588-s001.zip › Figure S5.pdf]

UMUC	0nM	1nM	10nM
mean	1	0.803239	0.502551

	5637 0nM	5637 1nM	5637 10nM
mean	1	0.292676	0.108111
