# Supplementary material for: The Role of FGFR3 in the Progression of Bladder Cancer
Source: Cancers (Basel). 2025 Nov 6;17(21):3588. doi: 10.3390/cancers17213588 (PMC12610005; doi:10.3390/cancers17213588)
Supplement: Supplementary file 1 [file cancers-17-03588-s001.zip › Figure S6.pdf]

| | UMUC si-c | UMUC si-FGFR3 |
|---|---|---|
| mean | 1 | 0.283752 |
| stdev | 0 | 0.065758 |

| | 5637 si-coi | 5637 si-FGFR3 |
|---|---|---|
| mean | 1 | 0.453612 |
| SD | 0 | 0.23834 |
