# Supplementary material for: The Role of FGFR3 in the Progression of Bladder Cancer
Source: Cancers (Basel). 2025 Nov 6;17(21):3588. doi: 10.3390/cancers17213588 (PMC12610005; doi:10.3390/cancers17213588)
Supplement: Supplementary file 1 [file cancers-17-03588-s001.zip › Figure S7.pdf]

		day1	day2	day3	day4	day5
UMUC si-control		1	1.115301	1.377237	1.403633	3.519512
UMUC si-FGFR3		1	0.986857	0.977199	0.976103	0.95564
UMUC STDEV		0	0.433397	0.537276	0.578174	1.50556
UMUC FGFR3 STDEV		0	0.081995	0.101316	0.109265	0.094035

		day1	day2	day3	day4	day5
	5637	1	1.865357	2.36622	2.438174	2.610526
5637 FGFR3		1	0.85057	0.824228	0.429749	0.414782
5637 STDEV		0	0.920629	1.18146	1.151658	1.153207
5637 FGFR3 STDEV		0	0.139171	0.222103	0.050653	0.061435
