# Supplementary figures and images for: The Role of FGFR3 in the Progression of Bladder Cancer
Source: Cancers (Basel). 2025 Nov 6;17(21):3588. doi: 10.3390/cancers17213588 (PMC12610005; doi:10.3390/cancers17213588)

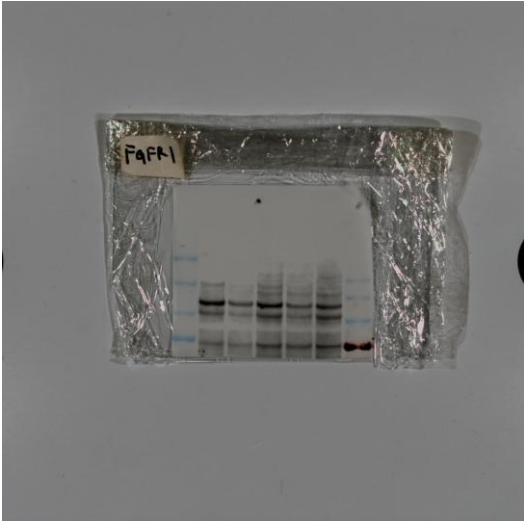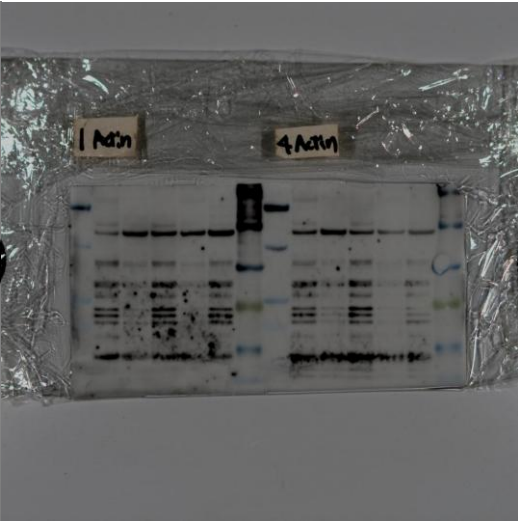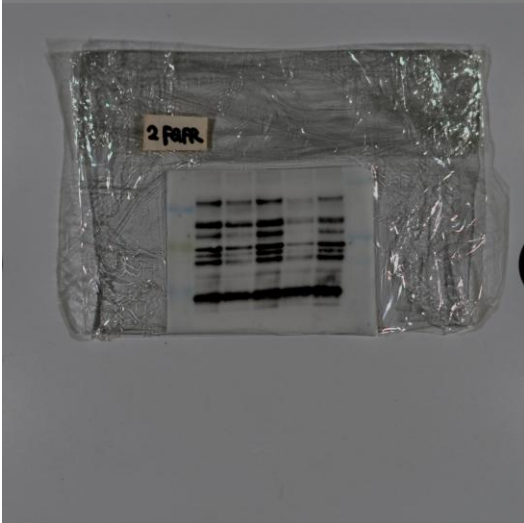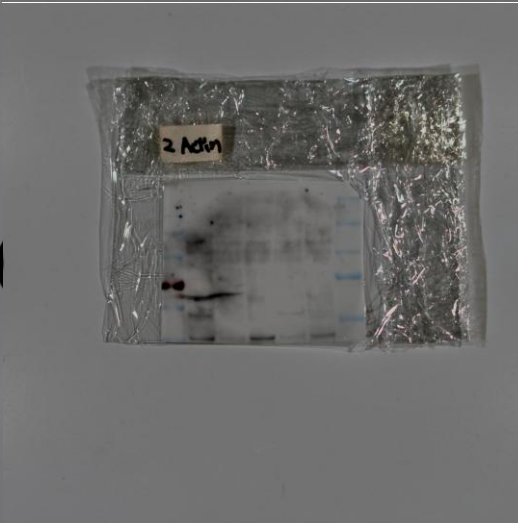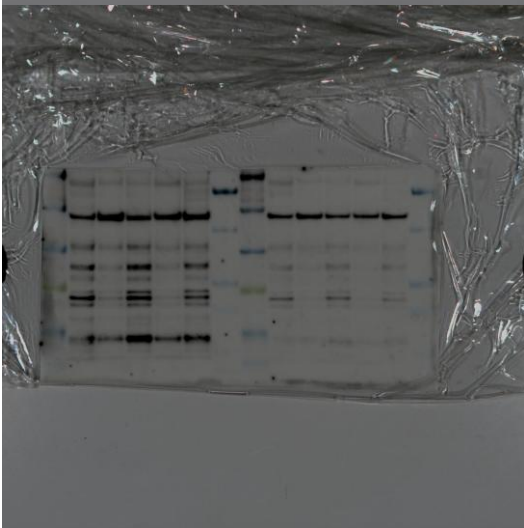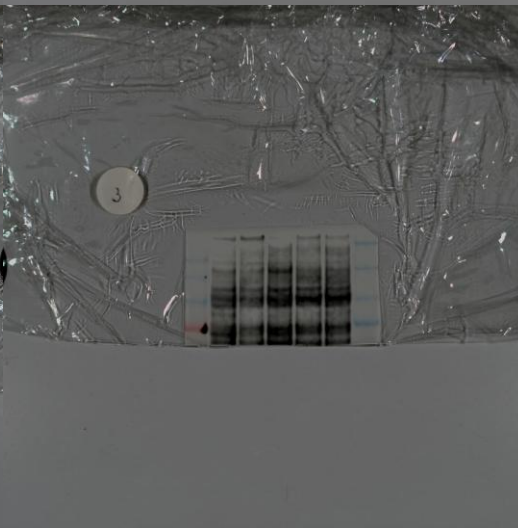

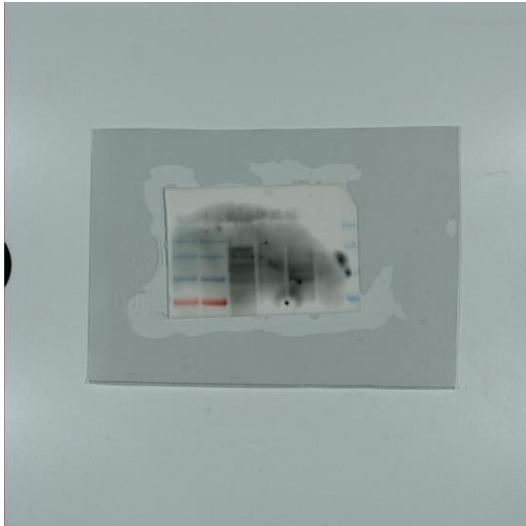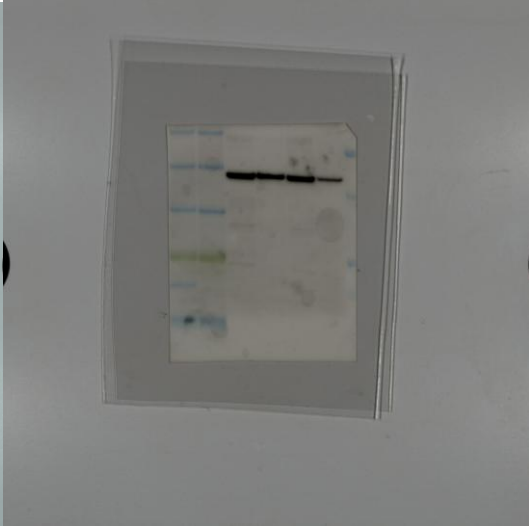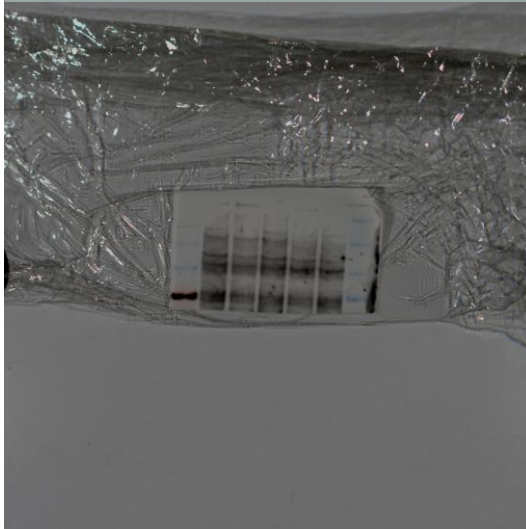

Supplement: Supplementary file 1 [file cancers-17-03588-s001.zip › Figure S1.pdf]
